# Supplementary material for: Expanded Newborn Screening in Italy Using Tandem Mass Spectrometry: Two Years of National Experience
Source: Int J Neonatal Screen. 2022 Aug 9;8(3):47. doi: 10.3390/ijns8030047 (PMC9397032; doi:10.3390/ijns8030047)
Supplement: Supplementary file 1 [file IJNS-08-00047-s001.zip › IJNS-1792935-SI.pdf]

International Journal of
Neonatal Screening

MDPI

Table S1. Cut off calculated on term infants aged 48-72 hours for biomarkers analyzed by a non derivatized method.

Carnitines	Number of laboratories	Minimum	Maximum	Median	Mean	SD	CV%
**C0 (Low) - free**	**13**	4.6	8.0	7.0	6.61	1.2	18.9%
**C0 (High) - free**	**13**	44.4	80.0	50.0	53.58	9.9	18.5%
**C2 (Low) - acetyl**	**13**	2.0	10.2	7.0	6.66	2.7	40.0%
**C2 (High) - acetyl**	**13**	38.0	88.0	50.0	54.4	12.5	22.9%
**C3 -propionyl**	**13**	3.00	6.00	4.70	4.26	1.00	23.5%
**C4 - butyryl**	**13**	0.55	1.00	0.92	0.86	0.15	18.0%
**C3DC+C4OH - sum of malonyl and 3-OH-butyryl**	**13**	0.34	0.59	0.50	0.47	0.08	16.2%
**C5 - isovaleryl**	**13**	0.22	0.85	0.40	0.44	0.19	44.2%
**C4DC+C5OH - sum of methylmalonyl and 3-OH-isovaleryl**	**13**	0.40	0.87	0.56	0.57	0.15	26.2%
**C5DC - glutaryl**	**12**	0.08	0.40	0.23	0.24	0.09	38.1%
**C5:1 - tiglyl**	**13**	0.02	0.11	0.05	0.05	0.02	48.8%
**C6 - hexanoyl**	**13**	0.08	0.60	0.17	0.19	0.13	68.9%
**C8 - octanoyl**	**13**	0.11	0.55	0.25	0.25	0.12	49.9%
**C10 - decanoyl**	**13**	0.17	0.53	0.25	0.29	0.10	33.7%
**C12 - dodecanoyl**	**13**	0.23	0.60	0.32	0.35	0.10	28.8%
**C14 - tetradecanoyl**	**13**	0.37	0.80	0.50	0.51	0.10	20.2%
**C14:1 - tetradecenoyl**	**13**	0.27	0.70	0.39	0.40	0.12	29.8%
**C16 (High) - palmitoyl**	**13**	5.60	9.00	7.23	7.35	0.90	12.2%
**C16 (Low) - palmitoyl**	**13**	0.48	1.68	1.00	1.13	0.43	38.2%
**C16:1 - hexadecenoyl**	**13**	0.41	0.80	0.52	0.54	0.11	20.7%
**C16OH - 3-hydroxypalmitoyl**	**13**	0.05	0.10	0.07	0.08	0.02	25.2%
**C16:1OH - 3-hydroxypalmitoleyl**	**13**	0.06	0.19	0.09	0.10	0.03	32.9%
**C18 (High) - octadecanoyl**	**13**	1.69	2.90	2.07	2.15	0.40	18.5%
**C18 (Low) - octadecanoyl**	**13**	0.09	0.51	0.35	0.36	0.12	33.9%
**C18:1 (High) - octadecenoyl**	**13**	2.43	4.00	2.90	2.98	0.45	15.1%
**C18:1 (Low) - octadecenoyl**	**13**	0.00	0.67	0.40	0.36	0.27	73.6%
**C18OH - 3-hydroxyoctadecanoyl**	**13**	0.03	0.08	0.04	0.05	0.02	33.5%
**C18:1OH - 3-hydroxyoctadecenoyl**	**13**	0.04	0.70	0.07	0.15	0.20	134%
**ratio: C0/(C16+C18)**	**12**	8.40	31.0	14.0	15.8	7.32	46.2%
**ratio: (C16+C18:1)/C2**	**11**	0.32	1.00	0.44	0.53	0.24	45.1%
**Aminoacids**	**Numer of laboratories**	**Minimum**	**Maximum**	**Median**	**Mean**	**SD**	**CV%**
**Phe - phenylalanine**	**13**	67.0	120	100	99.4	17.0	17.1%
**Tyr - tyrosine**	**13**	165	300	250	240	34.2	14.2%
**Val - valine**	**13**	160	300	243	236	41.4	17.6%
**Xle - sum of isoleucine, leucine, alloisoleucine and OH-proline**	**13**	181	300	245	242	41.9	17.3%
**Met (high) - methionine**	**13**	24.3	45.0	37.0	36.9	6.34	17.2%
**Met (low) - methionine**	**13**	4.2	9.14	7.0	7.3	1.80	25.2%
**Cit (high) - citrulline**	**13**	23.8	90.0	35.0	39.6	16.4	41.3%
**Cit (low) - citrulline**	**13**	3.0	7.0	4.7	5.1	1.4	28.1%
**Arg - arginine**	**13**	21.0	80.0	35.0	41.2	14.9	36.1%
**Orn - ornithine**	**13**	150	332	235	236	58.6	24.9%
**Others**	**Numer of laboratories**	**Minimum**	**Maximum**	**Median**	**Mean**	**SD**	**CV%**
**Suac - succinylacetone**	**12**	0.41	2.00	1.35	1.33	0.54	40.5%
**ArgSucc - argininosuccinate**	**11**	0.50	1.20	1.00	0.85	0.25	29.3%

Table S2. Cut off calculated on term infants aged 48-72 hours for biomarkers analyzed by a derivatized method.

Carnitines	Number of laboratories	Mean
C0 (Low) - free	2	11.4
C0 (High) - free	2	63.5
C2 (Low) - acetyl	2	5.5
C2 (High) - acetyl	2	58.0
C3 -propionyl	2	3.78
C3DC - malonyl	2	0.19
C4 - butyryl	2	0.88
C4DC - methylmalonyl	2	1.09
C4OH - 3-OH-butyryl	2	0.48
C5 - isovaleryl	2	0.37
C5DC - glutaryl	2	0.18
C5OH -3-OH-isovaleryl	2	0.43
C5:1 - tiglyl	2	0.08
C6 - hexanoyl	2	0.15
C8 - octanoyl	2	0.21
C10 - decanoyl	2	0.29
C12 - dodecanoyl	2	0.47
C14 - tetradecanoyl	2	0.53
C14:1 - tetradecenoyl	2	0.37
C16 (High) - palmitoyl	2	6.97
C16 (Low) - palmitoyl	2	1.13
C16:1 - hexadecenoyl	2	0.50
C16OH - 3-hydroxypalmitoyl	2	0.09
C16:1OH - 3-hydroxypalmitoleyl	2	0.14
C18 (High) - octadecanoyl	2	1.86
C18 (Low) -octadecanoyl	2	0.4
C18:1 (High) - octadecenoyl	2	2.82
C18:1 (Low) - octadecenoyl	2	0.20
C18OH - 3-hydroxyoctadecanoyl	2	0.06
C18:1OH - 3-hydroxyoctadecenoyl	2	0.07
ratio: C0/(C16+C18)	2	20.4
ratio: (C16+C18:1)/C2	2	0.33
**Aminoacids**	**Number of laboratories**	**Mean**
Phe - phenylalanine	2	80.0
Tyr - tyrosine	2	216
Val - valine	2	194
Xle - sum of isoleucine, leucine, alloisoleucine and OH-proline	2	197
Met (high) - methionine	2	32.0
Met (low) - methionine	2	8.60
Cit (high) - citrulline	2	29.0
Cit (low) - citrulline	2	5.60
Arg - arginine	2	26.7
Orn - ornithine	2	191
**Others**	**Number of laboratories**	**Mean**
Suac - succinylacetone	2	1.62
ArgSucc - argininosuccinate	2	1.14
